# A benign teratoma presenting as an obstruction of the nasal cavity: a case report

**DOI:** 10.1186/1752-1947-6-147

**Published:** 2012-06-12

**Authors:** Ibrahim Cukurova, Murat Gumussoy, Aytekin Yaz, Umit Bayol, Orhan Gazi Yigitbasi

**Affiliations:** 1 Department of Otolaryngology and Head and Neck Surgery, the Ministry of Health Izmir Tepecik Training and Research Hospital, Izmir, 35120, Turkey; 2 Department of Pathology, the Ministry of Health Izmir Tepecik Training and Research Hospital, Izmir, 35120, Turkey

## Abstract

**Introduction:**

Teratoma refers to a neoplasm that recapitulates all three germ layers. Teratomas may be histologically mature and oncologically benign. Teratomas may also be histologically immature while being oncologically benign, or they may harbor malignant components and have the potential to exhibit an aggressive biological behavior. Teratomas of the head and neck are extremely rare and usually present in the neonatal period. As a general rule, pediatric teratomas of the head and neck tend to be oncologically benign, whereas adult teratomas tend to be histologically and oncologically malignant. Most of these teratomas are found in the cervical region and nasopharynx. Calcification within the mass is often evident.

**Case presentation:**

A 27-year-old Caucasian man complaining of a nasal obstruction was admitted to our clinic in January 2006. A transnasal endoscopic examination revealed a mass arising from the nasal septum which was completely removed using an endoscopic approach. Histologically, it was determined to be a benign teratoma.

**Conclusion:**

Herein, we present a rare case, along with a review of the related literature, in order to emphasize that a benign teratoma of the nasal septum should not be ignored.

## Introduction

Benign teratomas are solid and cystic tumors composed of a variety of both immature and mature tissues derived from all three germ layers. The epithelial component of a benign teratoma usually consists of mature squamous epithelium and immature intestinal or respiratory epithelium. Primitive neuroepithelium with rosettes, pseudorosettes or neurofibrillary matrix predominates in some tumors. Pigmented retinal epithelium can also be seen. The mesodermal component consists of fibroblasts and embryonic, immature spindle cells embedded in a myxoid matrix. Islands of cartilage, smooth muscle cells, and skeletal muscle cells exhibiting varying degrees of maturity may also be present.

Teratomas of the head and neck are extremely rare and usually seen during the neonatal period. While pediatric teratomas of the head and neck tend to be benign tumors, adult teratomas tend to be histologically and oncologically malignant. Calcification within the mass is often evident. Sinonasal masses present with nasal obstruction symptoms. The presenting symptoms are those associated with a sinonasal mass. Radiologically and grossly, these tumors are heterogeneous and composed of solid and cystic components [1].

The following should be considered in the differential diagnosis of sinonasal teratomas: immature teratomas, teratomas with malignant transformation, sinonasal yolk sac tumors, sinonasal teratocarcinosarcomas, dermoid cysts, hamartomas, and hairy polyps. While teratomas can be surgically resected, malignant teratomas also require adjuvant radiotherapy [2].

## Case presentation

**Figure 1:**
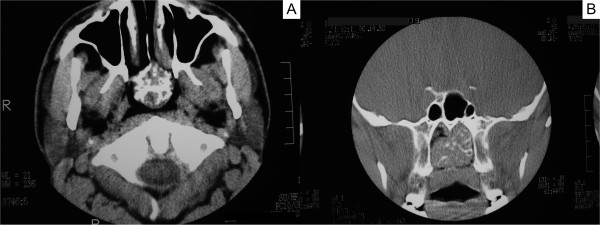
**(A and B) Axial and coronal computed tomography sections.** No adipose tissue or cystic components were seen on the computed tomography (CT) scan.

A 27-year-old Caucasian man who had been suffering from a nasal obstruction for eight years was admitted to the Department of Otolaryngology and Head and Neck Surgery, the Ministry of Health Izmir Tepecik Training and Research Hospital in January 2008. His past medical and family history did not include any abnormal or notable features. A mass with smooth surface, arising from the left posterior side of the nasal septum and filling the whole nasopharyngeal cavity, was seen on his endoscopic nasal examination. Other systemic examinations were normal. Complete blood count (CBC), activated partial thromboplastin time (APTT), and partial thromboplastin time (PTT) were normal. A soft tissue mass with lobulated margins was seen on computed tomography (CT) scan. Calcification was present in the septa. The mass extended from the posterior nasal septum to the pharyngeal space on the right and the nasal turbine on the left (Figure 1A and 1B). A punch biopsy was performed and the biopsy specimen was reported as sinonasal papilloma.

**Figure 2:**
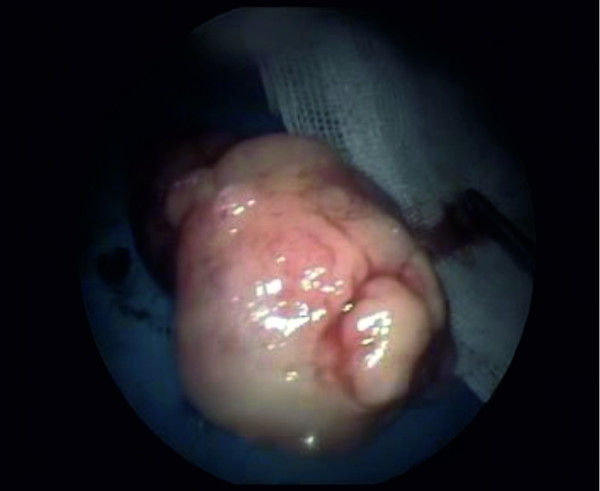
**Photograph of the nasopharyngeal mass.** The nasopharyngeal mass was approximately 4 cm × 3 cm in size and was totally resected under local anesthesia via an endoscopic approach.

The nasopharyngeal mass, approximately 4cmx3cm in size, was completely resected under local anesthesia via an endoscopic approach within our clinic (Figure 2). No complications were seen during the early postoperative period.

**Figure 3:**
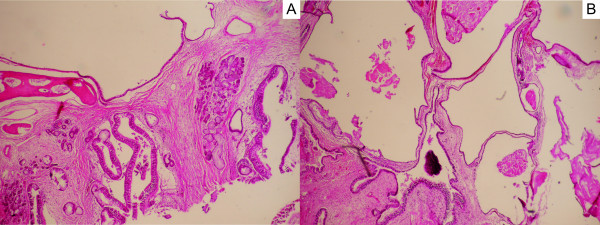
**Histology of the nasopharyngeal mass.** (**A**) Hamartomatous appearance resembling upper airway epithelium and consisting of septum bone frame, mucosa, mucosa-associated glands, and loose connective tissue stroma (hematoxylin and eosin (H&E), X40). (**B**) An epithelial component, loose connective tissue, and stroma-forming cystic structures were evident, partially covered with epithelial cells (H&E, X40).

Histopathological examination with hematoxylin and eosin staining revealed a semi-cystic nasal teratoma consisting of mature epithelial and mesenchymal components (Figure 3A and 3B).

The patient was followed up by periodic examinations for eight months and no recurrence was seen during this postoperative period.

## Discussion

Teratoma refers to a neoplasm that recapitulates all three germ layers. Teratomas of the head and neck are extremely rare and usually seen during the neonatal period. In general, lesions in the head and neck region account for approximately 5% of all benign and malignant germ cell neoplasms. Common sites of involvement include the neck, oropharynx, nasopharynx, orbit, and paranasal sinuses [3]. Mature teratomas of the sinonasal tract are even less common. Most of the cases occur in neonates and older infants, with equal distribution between girls and boys. In the sinonasal tract, the maxillary antrum and nasal cavity are affected more often than the sphenoid sinus. Common manifestations of mature teratomas include facial deformity, nasal obstruction, and a nasal mass [4].

Teratomas may be histologically mature and oncologically benign. Teratomas may also be histologically immature while being oncologically benign, or they may harbor malignant components and have the potential to exhibit an aggressive biological behavior. As a general rule, while pediatric teratomas of the head and neck tend to be oncologically benign, adult teratomas tend to be histologically and oncologically malignant [1,4].

The following should be considered in the differential diagnosis of sinonasal teratomas: immature teratomas, teratomas with malignant transformation, sinonasal yolk sac tumors, sinonasal teratocarcinosarcomas, dermoid cysts, hamartomas, and hairy polyps. Immature teratomas and teratomas with malignant transformation are tumors of infancy and early childhood, whereas sinonasal yolk sac tumors and sinonasal teratocarcinosarcomas have only been documented in adults [4-6]. Immature teratomas of the sinonasal tract are rare. Immature teratomas tend to be either solid-nodular or solid-cystic, while mature teratomas are usually cystic. Teratomas with malignant transformation are neoplasms composed of benign tissue elements of all three germinal layers, but which also have acquired somatic malignancy. Sinonasal yolk sac tumors are neoplasms with histological features of the embryonic yolk sac [5,6].

Sinonasal teratocarcinoma has a marked male predominance. These neoplasms almost exclusively arise in the ethmoid sinus and maxillary antrum. Patients usually present with a short history of nasal obstruction and epistaxis. Imaging studies reveal a nasal mass, occasionally accompanied by opacification of the paranasal sinuses. Bone destruction may be seen. Teratocarcinosarcomas are highly malignant. They are locally aggressive, rapidly invading soft tissue and bone as well as the orbit and cranial cavities. They also have the potential to metastasize to regional lymph nodes and distant sites, mainly the lungs. A dermoid cyst is a developmental lesion histogenetically and histologically composed of ectoderm and mesoderm, but not endoderm. Dermoid cysts of the nose account for 3% of all dermoid cysts and about 10% of those of the head and neck region. Dermoid cysts of the head and neck are located more often in the subcutaneous tissue of the lateral supraorbital ridge and nose. In the nose, they occur most commonly in the bridge and always in the midline. A few cases of dermoid cysts arising from the paranasal sinuses have been reported. The term hamartoma refers to an abnormal, oncologically benign proliferation of indigenous tissues. The sinonasal tract, particularly the area of the nasal septum, is the most common site for hamartomas of the head and neck region. At the histological level, benign disorganized epithelium, salivary glands, muscle, and cartilaginous and vascular tissues are seen [4-7].

The ideal treatment for mature teratomas is total surgical excision. Sinonasal teratomas are associated with high mortality rates, despite the utilization of different modalities in the treatment.

In our case, histopathological examination revealed epithelial and mesenchymal components, and the diagnosis of benign teratoma was established based on these findings. In the literature, benign teratomas arising from the nasal septum have only been described once in a young adult [8].

## Conclusion

The ideal treatment for mature teratomas is total surgical excision. Sinonasal teratomas are associated with high mortality rates, despite the utilization of different modalities in the treatment. We present a rare case, along with a review of the related literature, in order to emphasize that a benign teratoma of the nasal septum should not be ignored.

## Consent

Written informed consent was obtained from the patient for publication of this case report and any accompanying images. A copy of the written consent is available for review by the Editor-in-Chief of this journal.

## Competing interests

The authors declare that they have no competing interests.

## Authors’ contributions

IC performed the diagnosis of the patient and performed the operation. MG and AY collected data and performed statistical analysis. ÜB performed the histopathological examination. OGY had a major contribution in writing the manuscript. All authors read and approved the final manuscript.
